# HelloType1 Digital Education Platform for Individuals With Type 1 Diabetes in Southeast Asia: Data Analytics Study

**DOI:** 10.2196/91130

**Published:** 2026-07-16

**Authors:** Sze May Ng, Soe Nyi Nyi, Muhammad Yazid Jalaludin, Azriyanti Anuar Zaini, Phat Tung Ma, Sirimon Reutrakul, Taninee Sahakitrungruang, Sum Satha, Candy Gan, Darren Jia Chen Toh, Anne-Charlotte Ficheroulle

**Affiliations:** 1 Faculty of Health, Social Care and Medicine, Edge Hill University, St Helens Rd, Ormskirk, England, L39 4QP, United Kingdom, 44 1695656163; 2 Action4Diabetes, Somerset, United Kingdom; 3 Department of Paediatrics, Faculty of Medicine, University of Malaya, Kuala Lumpur, Malaysia; 4 Department of Endocrinology, University Medical Centre, Ho Chi Min City, Vietnam; 5 Division of Endocrinology, Diabetes and Metabolism, University of Illinois Chicago, Chicago, IL, United States; 6 Thai Association of Diabetes Educators, Bankgkok, Thailand; 7 School of Global Health, Faculty of Medicine, Chulalongkorn University, Bangkok, Thailand; 8 Department of Endocrinology, Calmette Hospital, Phnom Penh, Cambodia; 9 TypeOne.sg, Singapore, Singapore; 10 University of Cambridge, Cambridge, England, United Kingdom

**Keywords:** diabetes, education, digital, education, analytics

## Abstract

**Background:**

Type 1 diabetes remains an underrecognized and challenging condition across Southeast Asia, where many countries face limited health care infrastructure, shortages of trained health care professionals, inconsistent access to diabetes education, and a lack of culturally appropriate resources in local languages. These barriers contribute to delayed diagnosis, suboptimal self-management, high rates of diabetic ketoacidosis, and inequities in care. During the COVID-19 pandemic, Action4Diabetes, a nonprofit organization working with local health care professionals and diabetes associations across Southeast Asia, developed HelloType1, a multilingual digital educational platform designed to improve awareness, education, and access to credible type 1 diabetes information. The platform was launched sequentially in Cambodia in 2021, Vietnam and Thailand in 2022, and Malaysia in 2023 through formal memorandums of understanding with local partners.

**Objective:**

This study aimed to evaluate the reach, platform usage, and online engagement of the HelloType1 digital educational platform across Southeast Asia between 2021 and 2024.

**Methods:**

Website analytics from Google Analytics 4 and Meta Business Suite metrics were descriptively analyzed to assess digital reach and engagement across countries. Metrics were compared over time and by country to examine patterns of platform uptake and user engagement.

**Results:**

HelloType1 demonstrated substantial growth between 2021 and 2024. Total unique website users increased from 1178 in 2021 to 40,361 in 2024, representing a marked expansion in regional reach. Pageviews rose from 4644 in 2021 to 83,689 in 2024, suggesting increasing content use and user platform engagement. By 2024, most website visits originated from organic search engines. Platform use was predominantly mobile-based, particularly in Vietnam, Thailand, and Malaysia, with the strongest engagement among adults aged 25 to 54 years. Facebook followers increased from 940 to 4553, and average engagement rates rose between 2022 and 2024. Cambodia achieved the highest number of Facebook interactions, whereas Thailand demonstrated the highest engagement rate. Content-level analysis showed that practical self-management topics, including blood glucose monitoring, insulin treatment, nutrition and exercise, complications, and emotional support, generated high levels of reach and interaction.

**Conclusions:**

HelloType1 demonstrates strong growth, mobile-first use, search-driven visibility, and engagement with practical self-management content. These findings support the feasibility and potential utility of a low-cost, multilingual digital education model, while future studies should evaluate its effects on knowledge, behavior, and clinical outcomes.

## Introduction

According to the International Diabetes Federation Atlas 2025, nearly 1 in 9 adults globally (589 million people) are living with diabetes, of whom an estimated 252 million remain undiagnosed, placing them at increased risk of preventable complications and early mortality. Importantly, approximately 3-quarters of all people living with diabetes reside in low- and middle-income countries (LMICs) [1], highlighting the disproportionate burden borne by resource-limited regions. Within Southeast Asia, the International Diabetes Federation estimates that over 1 million people are living with type 1 diabetes (T1D), including 29,676 children and young people younger than the age of 19 years in 2024, representing an almost 3-fold increase compared with estimates from 2022 [1,2]. These data underscore the rapidly growing and historically underrecognized burden of T1D in the region.

The prevalence and management of T1D in Southeast Asia present substantial and underrecognized public health challenges [3]. Many countries in the region are LMICs with limited health care infrastructure, lack of comprehensive national diabetes care programs, and a severe shortage of accessible educational resources in local languages. Access to essential components of T1D management, including insulin, blood glucose monitoring devices, and structured diabetes self-management education, remains inconsistent and is highly variable between and within countries [4].

Beyond epidemiological growth, systemic health care limitations significantly compromise outcomes for people living with T1D in Southeast Asia. Many countries rely heavily on out-of-pocket health care expenditures, with limited insurance coverage for chronic disease management. Families frequently bear the full financial responsibility for insulin, glucose monitoring supplies, clinic visits, and hospital admissions. Annual diabetes-related costs for families in the region often exceed US $1000, a substantial economic burden in LMIC contexts [5]. These constraints contribute to delayed diagnosis, fragmented follow-up, and alarmingly high rates of diabetic ketoacidosis (DKA) at presentation, reflecting persistent gaps in awareness, early detection, and education. Accurate national prevalence data for T1D in Southeast Asia remain scarce due to underdiagnosis, inconsistent reporting systems, and limited prioritization of noncommunicable diseases within health policy frameworks [5,6]. As a result, many children and young people with T1D receive care only in specialized urban centers or private facilities, which are inaccessible to large segments of the population. These challenges are compounded by a lack of culturally appropriate, credible diabetes education materials in local languages, further limiting the capacity of patients, families, and caregivers to effectively manage the condition [3,5].

Recognizing these gaps, Action4Diabetes (A4D), a UK-based nongovernmental organization, developed HelloType1, a digital educational platform in collaboration with local health care professionals [4,5]. Launched during the global COVID-19 pandemic in 2021, this initiative sought to leverage growing internet penetration and digital technologies to overcome traditional barriers to diabetes education and care. HelloType1 is a platform that consists of a website and Facebook pages in local languages dedicated to empowering people living with T1D, caregivers, and health care professionals. HelloType1 was introduced sequentially in Cambodia (March 2021), Vietnam (March 2022), Thailand (November 2022), and Malaysia (May 2023), formalized through memorandums of understanding (MOU) with relevant local diabetes associations, thereby ensuring the content’s clinical validity and cultural appropriateness [4]. The platform targets both T1D and their caregivers, as well as health care professionals. The T1D domain has three subcategories: (1) “T1D Basics” covering understanding T1D, blood glucose monitoring, insulin, hypoglycemia and hyperglycemia, sick day management, nutrition, exercise, complications, and emotional well-being; (2) “Life with T1D” consists of school, work life, relationships, which lifestyle, and T1D stories; and (3) “For Caregivers” equipped with information on coping with stress, relationships, school, empowering children, educational tools, a parent corner, and resources for those newly diagnosed [5]. The health care professional domain hosts (1) webinars where registrations for new webinars are accepted and recordings of past webinars are hosted, (2) essential resource information on T1D, (3) upcoming meetings of the Southeast Asia T1D network and recordings of past meetings, (4) scientific publications from the network, and (5) A4D News [6,7].

This study aimed to evaluate the digital reach, aggregate use, and online engagement of the HelloType1 digital educational platform across Southeast Asia from 2021 to 2024. Specifically, we analyzed website and social media analytics to assess patterns of user growth, traffic sources, device use, country-level uptake, and engagement with local-language T1D educational content. By examining these metrics, the study sought to determine whether a culturally adapted, multilingual digital platform can provide a scalable and accessible model for improving T1D education in resource-limited settings.

## Methods

### Overview

This study used a retrospective-descriptive analytics design to evaluate the reach, platform usage, and engagement of the HelloType1 digital educational platform between January 1, 2021, and December 31, 2024. The platform consisted of the HelloType1 website and associated country-specific Facebook pages, which provided T1D education in local languages for Cambodia, Vietnam, Thailand, Malaysia, and Myanmar. The launch dates varied by country: Cambodia launched in March 2021, Vietnam in March 2022, Thailand in November 2022, and Malaysia in May 2023, with subsequent platform expansion to include Myanmar-related content and regional outreach. Website data were obtained from Google Analytics 4, which was implemented on HelloType1.com to monitor aggregate platform activity. Data were extracted for each calendar year from 2021 to 2024. The website metrics collected included total users, percentage of returning visitors, total pageviews, pages viewed per session (where available), traffic acquisition source, country of access, user age group (where available), and device category, including desktop and mobile access. Traffic sources were categorized according to Google Analytics default channel groupings, which included direct access, organic search, social and display traffic, and referral traffic.

Social media data were obtained from Meta Business Suite for the official HelloType1 country Facebook pages. Facebook metrics were extracted for the same study period where available and included total followers, organic page reach, page visits, number of posts, post reach, user interactions, and engagement rate. Interactions included reactions, likes, comments, shares, and other recorded user engagement actions available through Meta Business Suite. Content-level Facebook data were grouped into predefined educational categories, including “What is T1D?,” blood glucose monitoring, insulin treatment, hypoglycemia and hyperglycemia, sick day management, nutrition and exercise, T1D complications, emotional support, and campaigns or awareness content.

### Data Analysis

All analytics data were exported by an authorized HelloType1 administrator with access to the website and Facebook page dashboards. Data were downloaded as Microsoft Excel files and reviewed by the study team. Extracted datasets were checked for completeness, duplicate entries, inconsistent date ranges, and implausible values before analysis. Country-level data were organized according to platform launch status and calendar year to account for differences in the duration of platform availability. Where data were unavailable for specific years or countries because a platform had not yet launched, these cells were recorded as missing rather than zero. No individual-level identifiable user data were collected, analyzed, or stored.

Data were analyzed descriptively using Microsoft Excel. Annual totals were calculated for website users, pageviews, Facebook followers, reach, visits, posts, and interactions. Percentage growth was calculated using the formula:

Growth percentage = [(current year’s value − previous year’s value) / previous year’s value]×100

For example, annual growth in website users was calculated between 2021 and 2022, 2022 and 2023, and 2023 and 2024. Country-level growth between 2023 and 2024 was calculated using a combination of website users and Facebook followers. The engagement rate was calculated as:

Engagement rate=total interactions / total reach×100

Device-use patterns were summarized by age group and country using available Google Analytics demographic and technology reports. Content-level reach was summarized by educational category and country to identify which types of T1D education generated the greatest audience exposure. Because the study used aggregate anonymized platform analytics, no personal identifiers, user accounts, IP addresses, or individual-level health data of patients with T1D were accessed. The HelloType1 website does not require user registration to access educational content, and therefore individual user journeys, repeat-user behavior, or linkage between website and Facebook activity could not be assessed. The analysis was therefore limited to aggregate indicators of digital reach and engagement rather than individual-level educational or clinical outcomes. The datasets analyzed in this study consisted of aggregate, anonymized website and social media analytics obtained from Google Analytics 4 and Meta Business Suite for the HelloType1 website and associated country-specific Facebook pages. No individual-level identifiable data, patient-level data, IP addresses, user accounts, or health records were collected, analyzed, or stored.

### Ethical Considerations

Ethical approval was obtained from UK Edge Hill University registration number ETH2324-0207. All data were handled in aggregate form and used solely for the evaluation of platform reach, platform usage, and engagement.

## Results

Table 1 shows that HelloType1.com usage in 2024 was predominantly mobile-based across all countries, particularly in Vietnam, Thailand, and Malaysia. Engagement was highest among adults aged 25 to 54 years, with Malaysia contributing the largest user volume, while Cambodia and Thailand showed strong uptake among younger and working-age adults. Usage in Myanmar was minimal and confined to younger adults, reflecting early-stage platform adoption.

**Table 1.** Demographic distribution of HelloType1.com users in 2024 across Cambodia, Vietnam, Thailand, Malaysia, and Myanmar, stratified by age group and device type (desktop vs mobile).

Age (y)	Cambodia, n			Vietnam, n			Thailand, n			Malaysia, n			Myanmar, n		
	Desktop	Mobile	Total	Desktop	Mobile	Total	Desktop	Mobile	Total	Desktop	Mobile	Total	Desktop	Mobile	Total
18‐24	26	54	80	54	60	114	62	51	113	158	299	457	—	12	12
25‐34	48	58	106	—^a^	107	107	189	297	486	97	687	784	—	—	0
35‐44	14	18	32	—	189	189	114	407	521	118	1252	1370	—	—	0
45‐54	—	—	—	—	120	120	244	305	549	113	824	937	—	—	0
55‐64	—	—	—	—	98	98	24	185	209	—	567	567	—	—	0
≥65	—	—	—	—	128	128	12	100	112	—	70	70	—	—	0
Total	88	130	218	54	702	756	645	1345	1990	486	3699	4185	0	12	12

a Not applicable.

Table 2 demonstrates substantial growth in HelloType1 platform’s reach and engagement between 2021 and 2024. Total unique users increased with a marked shift toward organic search traffic (23%-78%) and a more than 18-fold increase in pageviews, indicating improved visibility and content relevance. Concurrently, Facebook engagement strengthened, with followers rising, reflecting sustained community interaction and growing user engagement over time. Between 2021 and 2022, the platform experienced substantial 613% growth, primarily driven by launches in Vietnam and Thailand. The growth trajectory continued with an additional 34% increase from 2022 to 2023, following the launch in Malaysia. By 2024, user distribution was predominantly through searches, with a significant minority engaging via social media, such as Facebook, followed by direct access.

Table 3 illustrates marked geographic variation in HelloType1 platform uptake and content engagement across Southeast Asia. Between 2023 and 2024, overall platform usage increased with the largest growth observed in Malaysia and Thailand reflecting rapid uptake following the platform’s launch, while Cambodia maintained consistently high engagement with more modest growth due to earlier implementation. Cambodia recorded the highest number of Facebook followers and sustained engagement, whereas Vietnam demonstrated similar proportional growth.

Analysis of Facebook activity in 2024 (Table 4) showed robust content dissemination across the region with Cambodia achieving the highest total interactions, while Thailand demonstrated the highest engagement rate. Myanmar exhibited very high reach, driven by awareness-focused dissemination but comparatively low engagement rates, consistent with early-stage platform exposure.

**Table 2.** Insights from HelloType1.com and Facebook metrics from 2021 to 2024 (all countries).

Metrics	2021	2022		2023		2024	
	Actual	Actual	Growth vs 2021 (%)	Actual	Growth vs 2022 (%)	Actual	Growth vs 2023 (%)
Google Analytics 4 metrics							
Total unique users	1178	8396	613	11,277	34	40,361	258
Returning visitors (%)	18	15	—^a^	18	—	16	—
Traffic source							
Direct (%)	28	26	—	22	—	9	—
Social and display (%)	41	20	—	18	—	13	—
Search (%)	23	48	—	56	—	78	—
Referral (%)	8	6	—	4	—	0	—
Total pageviews, n	4644	26,000	460	41,260	59	83,689	103
Facebook metrics							
Total followers	940	1488	58	2932	97	4553	55
Total page reach (organic)	—	66,940	—	147,544	120	102,146	−31
Page visit	—	5132	—	25,811	403	44,363	72
Average engagement rate (%)	—	8	—	14	—	29	—

a Not applicable.

**Table 3.** HelloType1 user distribution by country in 2023 and 2024.

Country	Year 2023			Year 2024			
	Website users	Facebook followers	Total	Website users	Facebook followers	Total	Growth vs 2023 (%)
Cambodia	4099	1814	5913	4158	2453	6611	12
Vietnam	1207	509	1716	4257	633	4890	185
Thailand	1086	181	1267	11,091	238	11,329	794
Malaysia	1142	428	1570	16,838	604	17,442	1011
Myanmar	—^a^	—	—	346	625	971	—
Other countries^b^	3743	—	3743	3671	—	3671	–2
Total	11,277	2932	14,209	40,361	4553	44,914	216

a Not applicable.

b Other countries: includes all user connections from locations where HelloType1 has not yet been launched.

**Table 4.** HelloType1 Facebook page posts, reach, and engagement.

Country	Total posts	Total reach (R)	Total interactions (I)	Average reach per post	Average interest per post	Engagement rate (I/R; %)
Cambodia	123	151,641	7066	1233	57	4.7
Vietnam	102	20,646	1527	202	15	7.4
Thailand	96	6731	767	70	8	11.4
Malaysia	107	14,144	1105	132	10	7.8
Myanmar	29	367,158	4655	12,661	160	1.3
Regional	457	560,320	15,120	1226	33	2.7

Content-level analysis (Table 5) revealed that actionable self-management topics, including blood glucose monitoring, insulin treatment, nutrition and exercise, and T1D complications, consistently achieved higher reach than general awareness content across most countries. Emotional support content showed particularly strong engagement in Cambodia and Vietnam. Collectively, these findings indicate that locally adapted, practical diabetes education content may be effective in driving meaningful engagement across diverse socioeconomic and digital contexts in Southeast Asia [7].

**Table 5.** HelloType1 Facebook reaches by content category and by country.

Content category	Cambodia	Vietnam	Thailand	Malaysia	Myanmar	Regional total
What’s T1D^a^?	4286	1087	321	522	18,635	24,851
Checking BGM^b^	3127	1770	358	1029	94,931	101,215
Insulin treatment	5031	3490	1481	1466	76,150	87,618
Hypo or hyperglycemia	5573	1963	536	1228	30,678	39,978
Sick day management	2112	139	224	592	25,723	28,790
Nutrition and exercise	7458	2964	1666	3142	38,811	54,041
T1D complications	7118	1010	664	1351	79,643	89,786
Emotional support	18,515	4602	1223	3184	2130	29,654
Campaigns or awareness	98,421	3621	258	1630	457	104,387
Grand total	151,641	20,646	6731	14,144	367,158	560,320

a T1D: type 1 diabetes.

b BGM: blood glucose monitoring.

## Discussion

### Principal Findings

The digital approach adopted by HelloType1.com may help to address critical gaps in diabetes education in Southeast Asia. These advantages are consistent with previous studies highlighting the potential efficacy of digital health education in managing chronic conditions [8,9]. The success of HelloType1 in engaging users, particularly through organic searches and social media interactions, emphasizes the potential of digital platforms in low-resource settings. The improvement in average pages per session indicates users’ increasing comfort with digital resources and their preference for exploring comprehensive information online rather than isolated content pieces. This aligns with global trends toward digital transformation in health care education, recognized as a cost-effective and accessible method, particularly in LMICs [8-10]. Among Southeast Asian countries, resources for T1D vary. For instance, Thailand, as an upper middle-income country, provides access to insulin, blood glucose monitoring supplies, and diabetes self-management education through its universal health care system [11]. The significant uptake of HelloType1 in Thailand demonstrates that even in countries with established health care services, digital platforms can complement the national support system by offering accessible, trusted educational resources. Strategic partnerships with local diabetes associations via MOUs ensured content relevance and credibility, bolstering community trust and use. Furthermore, the interactive design of the HelloType1 platform likely contributed to user engagement. Educational formats including articles, animated videos, quizzes, and personal stories provided diverse learning opportunities that catered to various learning preferences, potentially increasing content retention and application. Previous research has indicated that diverse content formats can significantly enhance user experience and educational outcomes in digital health platforms [12,13]. Moreover, targeted content addressing psychosocial aspects of diabetes management, such as stigma and emotional support, was essential in fostering a supportive community environment. This holistic approach aligns with recommendations advocating comprehensive diabetes education that incorporates psychosocial care alongside clinical management [14].

The analytics also highlighted demographic trends, with younger users representing higher user segments. This demographic insight underscores the importance of tailoring content and outreach strategies to effectively engage key audiences. Digital educational platforms must continuously evolve to meet demographic shifts and emerging user needs, ensuring sustained relevance and impact. The digital education model adopted by HelloType1 is particularly relevant given the multifaceted challenges associated with managing T1D in Southeast Asia. Families face substantial economic burdens due to recurring outpatient visits, medications, and inpatient care, which can cumulatively exceed US $1000 annually in LMICs [15]. These direct and indirect costs often create barriers to consistent care and long-term adherence.

However, the burden extends beyond financial hardship. Children and adolescents with T1D require continuous psychosocial and behavioral support, which is frequently undermined by low awareness, cultural misconceptions, and inadequate health literacy [16]. Emotional stress, stigma, and lack of family understanding can further complicate diabetes management. Adolescents, in particular, face unique challenges as they seek independence, often experiencing communication breakdowns with caregivers [16-18]. Social contexts also play a role in diabetes mismanagement. Many families fear disclosing a child’s diagnosis due to perceived stigma, especially in the case of female patients with T1D, where marital prospects may be affected. Misconceptions about diet, exercise, and insulin use—along with unscientific practices such as reliance on herbal remedies—further endanger patient outcomes. These issues are compounded in schools, where teachers often lack the necessary knowledge to accommodate T1D [3].

The platform’s unique strength lies in its alignment with best practices for diabetes self-management education and support, as endorsed by the International Society of Pediatric and Adolescent Diabetes. By offering diverse, localized formats ranging from infographics and animations to live webinars and regional taskforce meetings, HelloType1 fosters a learning environment suited for both laypeople and health professionals [7]. This aligns with research showing that digital health interventions are more effective when they cater to users’ literacy levels, learning styles, and cultural expectations [10,13]. By offering accurate, local language digital resources, HelloType1 directly addresses many of these systemic, cultural, and psychosocial barriers. Its design aligns closely with global recommendations and integrates psychosocial care as an essential pillar of T1D management [19]. Additionally, growing evidence shows that well-designed digital platforms can improve diabetes knowledge, self-efficacy, and glycemic outcomes, particularly when the content is engaging and culturally tailored [20]. Interventions like HelloType1, which offer structured, multiformat education in local languages, reflect successful models from other regions and represent an important innovation for Southeast Asia. The platform’s demonstrated growth and engagement across multiple countries add to this evidence base, showing real-world success in extending digital diabetes education to underserved populations [7,21].

One of the most compelling implications of the HelloType1 platform is its potential to transform survival and self-management outcomes in LMICs. Before the implementation of A4D-supported interventions, Laos had no recorded long-term survivors of childhood T1D [22]. Survival became possible only after the introduction of A4D’s Clinic Support Programme in 2016, which provided essential supplies and education. Although HelloType1 in the Laotian language has yet to be launched, by offering diabetes information in the local language in Laos and online access to psychosocial and practical guidance, HelloType1 can complement these clinical efforts with community-based empowerment [7,22]. While A4D has not launched a Laotian version of HT1, it is important to note that the Lao and Thai languages are highly similar and that many Laotians, especially those near the Thai border or in contact with Thai media, likely understand or speak some Thai [23]. Moreover, the platform reflects a broader shift toward rights-based digital health access. Insulin unavailability and prohibitive costs still plague most Southeast Asian countries [7,15]. The HelloType1 platform does not address these issues directly but mitigates their impact through knowledge dissemination. Education becomes a buffer against misinformation, taboos, stigma, and poor decision-making. When young people and caregivers are equipped with the knowledge to recognize symptoms early or challenge T1D management misconceptions, they are more likely to demand appropriate care and adhere to treatment regimens.

This approach is especially important in contexts where misconceptions and nonscientific remedies prevail. In the HelloType1 analytics, Vietnamese and Cambodian Facebook users, particularly younger adults, showed higher engagement with educational content, indicating a demand for culturally resonant and gender-sensitive information [7]. These findings align with broader global concerns raised by the Lancet Commission on Diabetes, which stresses the importance of using data to reform patient outcomes and ensure equitable care across regions [24]. HelloType1 represents a model for how digital tools, when informed by local epidemiology and user behavior, can serve not just as health platforms but as advocacy instruments. However, digital transformation cannot substitute for systemic change. Although HelloType1 is an essential educational tool, it remains limited in its ability to address structural barriers such as out-of-pocket payments, insulin shortages, and fragmented service delivery. As indicated in both the A4D Practical Diabetes paper [4] and the Diabetes Research and Clinical Practice article [25], rates of DKA at diagnosis remain worryingly high at 30% to 90% in many settings. These data suggest that the digital reach of HelloType1 needs to be complemented by in-person training, diabetic family camps, national policy reforms, and broader health system strengthening.

A deeper challenge remains regarding equitable access to digital platforms themselves. While HelloType1’s reach has increased significantly through organic search traffic and social media, it is important to recognize that internet penetration, especially in rural Southeast Asia, is not uniform. Many families with children living with T1D may not have regular access to smartphones, let alone sufficient data for streaming videos or downloading resources. While the Southeast Asian region’s average internet penetration is 67%, Vietnam (70.3%), Thailand (69.5%), and the Philippines (67.0%) have higher or equal coverage as compared to the regional average, whereas Cambodia (52.6%), Laos (48.4%), Timor-Leste (45.1%), and Myanmar (43.3%) have lower internet penetration than the regional average [25].

Although concrete data on the digital divide between urban and rural areas in Southeast Asian countries is not available, it can be considered that internet coverage in rural Southeast Asian areas is much lower than in urban areas. Although efforts have been made to design HelloType1 for mobile compatibility and low-bandwidth environments, further strategies may be required, such as offline functionality or printable resources, for communities with low digital literacy.

As in the case of Laos, geographical remoteness is also an important challenge in accessing health care [22]. Children living far from Vientiane had higher rates of being lost to follow-up or experiencing poor glycemic outcomes. These findings suggest that while digital platforms offer scalable benefits, they cannot fully replace decentralized in-person services. HelloType1 could therefore benefit from integration into blended educational models, pairing digital content with community-based peer support, family camps, and health worker outreach [7,13,22]. Furthermore, engagement does not equate to impact. Increased page views or Facebook likes do not inherently reflect improved diabetes knowledge or behavioral change. Evaluating HelloType1’s influence on clinical outcomes such as glycosylated hemoglobin (HbA_1c_) trends, DKA readmissions, or treatment adherence will require longitudinal studies. The preliminary insights from A4D-supported clinics in 5 Southeast Asian countries show average HbA_1c_ levels of 9.7% (83 mmol/mol) [25], which is above the international target of 7% (53 mmol/mol) in settings without advanced diabetes technologies, such as continuous glucose monitoring [26]. While HelloType1 may raise awareness, true transformation hinges on whether families feel supported enough to apply the knowledge amid socioeconomic constraints.

In addition, HelloType1’s partnership-based development process, grounded in MOUs with local hospitals and national diabetes associations, enhances its legitimacy and sustainability. Unlike top-down interventions, this collaborative model ensures continuous feedback and context-specific updates. For instance, the localization of content to include caregiver support groups and country-specific dietary guidance has likely contributed to the observed engagement spikes in Vietnam and Cambodia [7]. Nevertheless, further research should examine how different user groups, such as people living with T1D, health care providers, and caregivers, use and interpret HelloType1 content. Health literacy is not a fixed trait, but an evolving practice shaped by social context, trust, and cultural norms [27,28]. Understanding users’ lived experiences would thus enhance the platform’s capacity to serve as both an educational and social support hub. Looking ahead, HelloType1 may also consider expanding its presence across alternative platforms. Messaging services such as Telegram or WhatsApp are widely used in Southeast Asia and offer a more intimate, conversational format that could enhance user retention and responsiveness. Similarly, short-form video content via TikTok or YouTube Shorts may appeal to adolescent audiences. As the digital landscape evolves, HelloType1 must remain agile, adaptive, and inclusive in its strategies.

### Limitations

This study has several limitations. First, the analysis relied on routinely collected aggregate digital analytics from Google Analytics 4 and Meta Business Suite. These platforms provide useful indicators of reach and engagement, but their metrics are influenced by platform-specific algorithms, changes in data definitions over time, browser restrictions, cookie settings, and user privacy controls. Users accessing the website from different devices, locations, or browsers may therefore have been counted more than once, while others may not have been fully captured.

Second, the data were limited to website and Facebook activity. Other digital channels, including messaging applications, Instagram, YouTube, TikTok, Telegram, WhatsApp, LINE, Zalo, and offline sharing of resources, were not included. This may underestimate the wider dissemination and use of HelloType1 content, particularly in Southeast Asian countries where messaging platforms are widely used for health communication. Third, engagement metrics such as pageviews, reach, likes, shares, comments, and interactions provide information on user activity but do not directly measure learning, behavioral change, diabetes self-management, or clinical outcomes. Therefore, although increased reach and engagement suggest growing visibility and acceptability, this study cannot determine whether HelloType1 improved diabetes knowledge, treatment adherence, glycemic control, DKA rates, or psychosocial outcomes.

Country-level differences may also reflect variations in promotional activity, social media use, internet access, smartphone availability, digital literacy, health literacy, local partnerships, and national diabetes service infrastructure rather than differences in platform effectiveness alone.

It is also noted that digital access remains unequal across Southeast Asia, particularly in rural, remote, and socioeconomically disadvantaged communities. Families with limited internet connectivity or restricted access to smartphones may be underrepresented in the analytics. This means that the findings may overrepresent users who are already digitally connected and may not fully reflect the educational needs of the most underserved populations. Future development of HelloType1 should consider qualitative methods such as focus group discussions or in-depth interviews, which will provide valuable contextual understanding to complement the quantitative metrics and support a user-centered evaluation [29]. Incorporating preintervention and postintervention assessments in future research is essential to establish educational effectiveness [30,31]. Language and literacy variations within each country were also not accounted for in the analytics.

Despite providing content in local languages, Southeast Asia is highly diverse, with varying levels of health literacy and digital literacy. This heterogeneity may hinder equitable access to online educational materials, especially among rural and socioeconomically disadvantaged populations [27,28]. Such contextual differences may limit the comparability of outcomes across countries and must be interpreted with caution [32]. The study focused on website and Facebook analytics, excluding other increasingly influential digital platforms, such as Instagram, YouTube, and messaging applications, which are known to play vital roles in health communication, especially in low-resource settings [33,34]. Direct messaging applications may represent another critical component of digital health communication strategies in Southeast Asia, with platform popularity varying considerably across countries. WhatsApp is widely utilized in Indonesia, Malaysia, and Singapore, while LINE holds a dominant position in Thailand. Facebook Messenger remains the most frequently used platform in the Philippines, whereas Zalo is the leading service in Vietnam, supporting both personal and business communication. Telegram has gained prominence in Cambodia and Myanmar, particularly in contexts requiring large group engagement and enhanced privacy features. Viber, though less regionally dominant, is extensively used in the Philippines and is present in other Southeast Asian countries.

### Conclusions

HelloType1 has demonstrated a potential role for digital health education platforms tailored to local contexts in Southeast Asia. Beyond the metrics, HelloType1 also highlights how context-specific digital innovations may help address deeper structural and psychosocial barriers to T1D. By offering local language content and targeting both people living with T1D and their caregivers, the platform responded to widespread challenges such as disease awareness, stigma, and limited school or community support. Its integration of diverse formats, including articles, videos, webinars, and caregiver-specific content, reflects best practices in digital education and may support accessibility for users with varying literacy levels and learning preferences. As the platform continues to evolve, future efforts should explore its impact on long-term diabetes self-management behaviors and health outcomes, while addressing access gaps and advancing multiplatform strategies to extend its reach. Future developments may also consider the integration of AI-driven chatbots within these platforms, which may expand the scalability, interactivity, and effectiveness of initiatives such as HelloType1.

## Abbreviations

A4D: Action4Diabetes
DKA: diabetic ketoacidosis
HbA_1c_: glycosylated hemoglobin
LMIC: low- and middle-income country
MOU: memorandum of understanding
T1D: type 1 diabetes
